# Clinicians’ satisfaction with laboratory services and associated factors at public health facilities in Northeast Ethiopia

**DOI:** 10.1186/s12913-023-09429-0

**Published:** 2023-05-11

**Authors:** Daniel Dagne Abebe, Minwuyelet Maru Temesgen, Addisu Tesfie Abozin

**Affiliations:** Amhara Public Health Institute Dessie Branch, Dessie, Amhara region Ethiopia

**Keywords:** Medical laboratories, Clinician satisfaction, Customer experience, Factors, Objective quality measures, Ethiopia

## Abstract

**Background:**

Satisfaction has become a key measure of quality and an important tool for improvement. Laboratories are increasingly required to regularly assess satisfaction of their customers. This study aimed to assess clinicians’ satisfaction with laboratory services and associated factors at public health facilities.

**Methods:**

A facility-based cross-sectional study was conducted in Northeast Ethiopia from May to June 2019. Eight hospitals and 24 health centres were first selected using a stratified sampling method, and a total of 224 randomly selected clinicians were included. Satisfaction with multiple aspects of laboratory services was assessed using a self-administered questionnaire, on a rating scale of 1 (very dissatisfied) to 5 points (very satisfied). Laboratory quality assessment was performed using WHO-AFRO’s stepwise accreditation checklist. Multivariable logistic regression model was fitted to determine the association between independent variables and clinicians’ overall satisfaction level using STATA ver14.1. A *p*-value < 0.05 was considered significant.

**Results:**

Overall, 72.8% of the clinicians were satisfied. Lowest mean ratings were obtained for the helpfulness of the laboratory handbook (3.3), provision of STAT/urgent services (3.7), and adequacy of tests provided (3.8). The clinicians’ timely receipt of results (AOR = 2.3, 95% CI = 1.1–5.0), notification of panic results (AOR = 2.5, 95% CI = 1.1–5.6), perceived quality/reliability of test results (AOR = 3.1, 95% CI = 1.5–6.3), and the laboratories’ rate of concordant malaria microscopy results (AOR = 4.1, 95% CI = 1.8–9.3), were significantly associated with satisfaction.

**Conclusions:**

Nearly one-third of clinicians were not satisfied with the laboratory services. Laboratory managers should emphasize the timely communication of STAT/urgent and panic results, and the reliability of test results, to improve users’ satisfaction and overall quality of care.

## Background

Medical laboratories are an essential component of effective healthcare system. Laboratory results must be accurate to ensure that subsequent medical decisions will lead to the best possible patient outcomes [1]. Results should also be delivered on time, as physicians could prefer empirical diagnosis to delayed diagnosis [2]. However, because access to quality testing is severely limited or undervalued, misdiagnosis commonly occurs in sub-Saharan Africa [1, 3].

Quality is providing excellent care as per published norms, thereby meeting customers’ needs and expectations [4, 5]. Customers’ satisfaction is a key element of quality and an important tool for improvement [5]. Clinicians are the primary customers of laboratories, and obtaining their feedback provides opportunities to identify gaps [6]. Now a days, even the most technically competent care is meaningless if unacceptable to users [5]. Satisfied clinicians are more likely to use laboratory tests routinely. Accreditation standard also emphasize customer satisfaction as a requirement for quality and competence [7, 8].

In Ethiopia, the laboratory structure is integrated with the healthcare tier, which includes health centres, and primary, general and specialized hospitals [9]. The country has made significant advances in expanding access to healthcare [10]. There have been substantial efforts aimed at improving quality, including the WHO-AFRO’s stepwise accreditation of laboratories [8, 10]. However, the achievements thus far remain inconsistent and the actual impact on users’ outcomes largely remains unclear [11, 12]. Customers’ satisfaction enables to link the current status of quality improvement with real customers’ expectations [6, 13]. Laboratories are also expected to regularly assess satisfaction to maintain accreditation, but not common in low-income settings [7, 8].

Many aspects of laboratory services could be investigated from the clinicians’ perspective, such as the availability of ordered tests, courtesy and respect, report format, turnaround time (TAT), critical result notification, and reliability [5, 6]. Previous studies have shown that physicians were most dissatisfied with the timeliness of results, advisory services, notification of panic values [14–17], and behavioural manners [14, 18, 19]. However, those studies have rarely explored satisfaction in relation to objective measures of laboratory practice [11, 14, 20, 21]. Many also argue the validity of user satisfaction as a measure of quality, particularly technical aspects, as users might be more sensitive to behavioural aspects [18, 19].

Assessment from multiple perspectives could provide a better basis to inform opportunities for balanced and thus sustainable improvement [5, 6, 20]. Therefore, this study aimed to assess clinicians’ satisfaction with laboratory services provided and its associated factors at public health facilities in Northeast Ethiopia.

## Methods

### Study design and area

A facility-based cross-sectional study was conducted from May to June 2019 in East Amhara, Northeast Ethiopia. This area covers six of the 15 zones of Amhara region. There were 402 governmental health facilities, of which only 252 facilities (35 hospitals and 217 health centres) were diagnostic. The laboratories provide basic tests such as serology, urinalysis, parasitology, malaria, tuberculosis (TB) microscopy and Gram staining [9]. The hospital levels additionally provide more advanced tests, such as fully automated clinical chemistry, CD4 count, electrolyte, hormone analysis, and microbiology tests. Amhara Public Health Institute Dessie Branch (APHI-DB) coordinates capacity building and external quality assurance (EQA) activities for the laboratories in the area. There were about 4,806 health professionals (physicians, health officers and nurses) in the study area, according to the Amhara region’s 2017 annual performance report.

### Source population

All clinical service providers who were working at governmental health facilities of East Amhara, Northeast Ethiopia, were the source population.

### Study population and eligibility criteria

All clinicians who were using laboratory services at the randomly selected public health facilities and were on duty during the study period were the study population. Clinicians who worked in the facility for shorter than six months were excluded.

### Sample size and sampling procedure

Eight hospitals and 24 health centres were included, accounting for 25% of the hospitals and 12.5% of the health centres in the study area. We did not cover the recommended sample size for health centres (25–30% based on the common rule-of-thumb), due to feasibility reasons.

The sample size of clinicians was determined using OpenEpi ver3.03 with the common formula for a single population proportion and applying a finite population correction: n = [N*p(1−p)]/ [(d2/Z21−α/2)*(N−1)+ p*(1−p)]. Considering a 95% confidence level (*Z*_1−α/2_ = 1.96), *p* = 80.0% from a study conducted in Ethiopia [17], and a margin of error of d = 5%. Therefore, considering a 10% nonresponse rate, the total sample size required was **n = 272 clinicians**.

The required sample size was then pre-allocated to each facility proportional to facility size but kept as fixed average quotas by facility type (for operational feasibility) – 10 clinicians from a hospital and eight from a health centre.

Facilities were selected using a stratified random sampling method with probability proportionate to facility size (client load) [22]. List frame of all facilities was first constructed stratifying by facility type/level in geographical order (providing implicit stratification), together with expected client loads. Systematic sampling was then employed to select the required number of facilities from the complete list frame at once. A single sampling interval (k) determined based on cumulative facility sizes was applied across all strata. This would ensure sufficient samples from small strata with few but large facilities while maintaining final data self-weighting. At each facility, clinicians were selected using simple random sampling from eligible clinicians on work at different clinical units.

### Study variables

The dependent variable was the overall satisfaction level of a clinician. It was measured based on satisfaction ratings towards multiple aspects of laboratory service (e.g., adequacy of test menu, STAT/urgent services). Independent variables include demographics (e.g., age, sex, professional category) and clinician-reported experiences (e.g., notified of panic results, perceived quality/reliability of results). Facility-level variables include objective key performance indicators (e.g., test availability, stepwise accreditation level, the accuracy of microscopy results).

### Data collection tools and procedures

A pre-tested, structured self-administered questionnaire was used to collect data from clinicians. It was customized for local use from a nationally validated tool for clinician customers of general laboratories in Ethiopia [14]. The questionnaire contained different questions related to demographic characteristics (eight), clinician-reported experiences of laboratory service (nine), and levels of satisfaction (nine). A five-point Likert rating scale ranging from “very dissatisfied” to “very satisfied” (1 to 5 points) was used for the nine satisfaction measuring items.

At the facility level, WHO-AFRO’s stepwise accreditation checklist was used to audit quality system practices and readiness of required tests [8, 9]. Blinded slide rechecking was also performed to evaluate the accuracy of microscopy results by systematically collecting slides examined in the previous quarter. Thirty malaria and 40 TB slides were rechecked per facility, following national EQA guidelines [21].

Data collectors and supervisors were senior laboratory experts recruited from external facilities. They were then trained on the data collection tools and procedures. All tools were pre-tested in nearby facilities before use for the actual data collection.

### Measurements

Satisfaction ratings given for the nine satisfaction measuring items were averaged to create an overall mean score for each respondent. Respondents with a score of ≥ 4 out of five points (i.e., combined very satisfied and satisfied ratings) were classified as satisfied, while the rest were classified as not satisfied. For each laboratory, the availability of required tests was measured as a percentage of the standard test menu expected for the respective facility type [9]. The stepwise accreditation score was calculated as a percentage of points met out of the total points on the checklist (275 points), and stars were graded from zero (if score < 55%) up to five (if score ≥ 95%) [8]. Concordance rates for the microscopy results were calculated as a percentage of the correct readings of the total rechecked slides [21].

### Data quality assurance

Training was given to data collectors and supervisors on the recruitment of participants and data collection. Pre-tested, structured, and standardized tools were used to ensure consistent data collection. Certified external laboratory assessors conducted the laboratory assessments. Supervisors oversaw the data collection and checked the consistency and completeness of the completed questionnaires.

### Operational definition

***Satisfaction level*** is the degree of perception or feeling to which service quality attributes have fulfilled the customer’s needs and expectations [14]. It is acknowledged as an outcome measure of service quality in this study.

### Data management and analysis

Data were entered using EpiData ver3.1 and analysed using STATA ver14.1. Descriptive statistics (mean, SD, and percentages) were computed and compared by facility type. Stepwise logistic regression analysis was used to identify individual- and organizational-level factors affecting clinicians’ overall satisfaction level. Those variables with a *p*-value < 0.20 in the bi-variable analysis were considered to be included in the final multivariable model. A *p*-value < 0.05 was considered statistically significant. Adjusted odds ratios (AORs) with 95% confidence intervals (CIs) were reported.

### Ethical consideration

Ethical clearance was obtained from the regional Ethical Review Board of Amhara Public Health Institute (APHI) Head office, Bahir dar, Amhara region, Ethiopia. The respective zone health departments and facility administrators were informed about the general aim and significance of the study through an official permission letter. The purpose of the study was described to each eligible clinician and all voluntary participants gave informed consent before enrolment. Data were collected anonymously without personal identifiers to ensure confidentiality. The study complied with the Helsinki Declaration.

## Results

### Clinicians’ socio-demographic characteristics

A total of 224 clinicians participated in this study accounting for a response rate of 82.7%. The mean age (± SD) of the participants was 30.9 (± 8.6) years, and nearly two-thirds of them (66.5%) had work experiences of four and more years. Table 1 shows the background characteristics of the clinicians. Half (50.0%) of the hospital clinicians were medical doctors and specialists, while 87.1% were health officers and nurses in health centres.

**Table 1:** Clinicians’ socio-demographic characteristics, Northeast Ethiopia, 2019

Variable/Category	Number (n = 224)	Percent
Sex^#^		
Male	137	61.4
Female	86	38.6
Age group^#^		
18–24 years	33	16.8
25–34 years	113	57.7
35–40 years	22	11.2
> 40 years	28	14.3
Marital status^#^		
Single	106	48.0
Married	113	51.1
Divorced	2	0.9
Profession category		
HO^*^, Nurse	178	79.5
MD^^^, Specialist	46	20.5
Work experience^#^		
0.5-3 year	73	33.5
4–10 years	100	45.9
10–38 years	45	20.6
Clinical unit/ward		
Maternal, Chronic	16	7.1
Outpatient	140	62.5
Emergency	19	8.5
Other	49	21.9
Facility type		
Hospital	46	20.5
Health centre	178	79.5

^*^HO = health officer; ^^^MD = medical doctor.

^#^There was one missing data for sex, 3 for marital status, 6 for work experience and 28 for age.

### Clinician-reported experiences of laboratory services

Three-fourths of clinicians reported the absence of a laboratory handbook (75.1%). Nearly one-third claimed to receive results out of the expected TAT (34.4%), poor quality/reliability of test results (35.3%), and lack of backup/specimen referral system (42.5%). Compared to health centres, more percentages of hospital clinicians reported poorer experiences on the timely receipt of results, notification of panic results and quality/reliability of test results (Table 2).

**Table 2:** Clinician-reported experiences of laboratory services, Northeast Ethiopia, 2019

Clinician-reported experience *(“Yes”)*	Health centre (n = 178)		Hospital (n = 46)		Overall (n = 224)	
	*n*	*%*	*n*	*%*	*n*	*%*
Laboratory handbook available	45	25.7	10	21.7	55	24.9
Personnel available whenever needed	159	91.9	37	80.4	196	89.5
Received test results within TAT^*^	115	70.1	22	48.9	137	65.6
Panic results communicated timely	137	79.2	24	52.2	161	73.5
Notified newly introduced tests always	66	37.9	9	19.6	75	34.1
Notified service interruptions always	71	41.0	13	28.9	84	38.5
Quality/reliability of results consistent	119	67.6	24	53.3	143	64.7
Backup/specimen referral available	97	66.0	14	30.4	111	57.5
Backup/referral test reports are reliable	96	65.8	20	47.6	116	61.7

*TAT indicates turnaround time.

### Characteristics of the participating laboratories

**Fig. 1:**
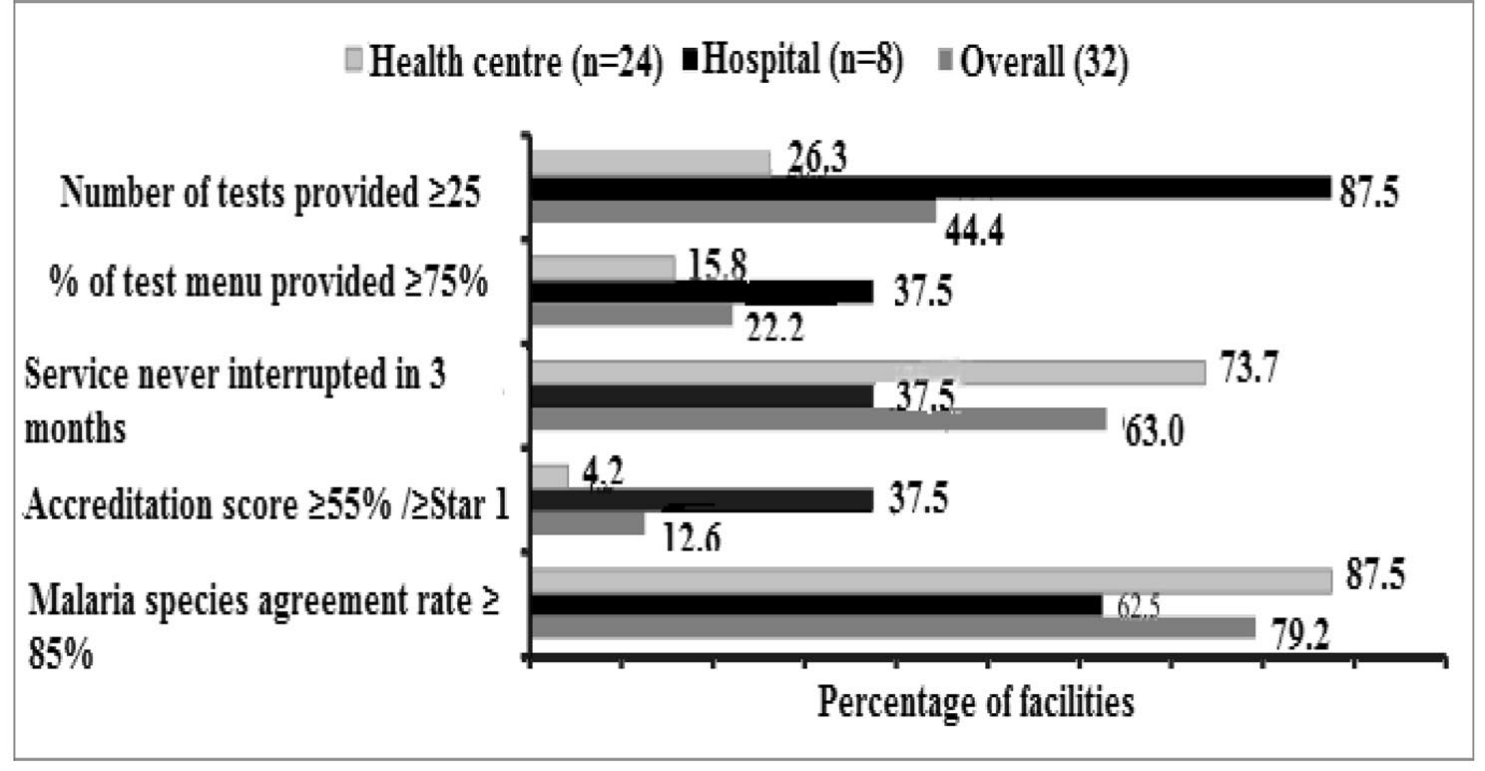
Percentages of laboratories with high-performance levels by facility type, Northeast Ethiopia, 2019

This study included eight hospitals (25.0%) and 24 health centres (75.0%). The total number of tests provided increased with an increase from the health centre to hospital levels. The overall mean scores observed were generally poor, such as for test availability (53.6% ±16.3) and quality systems (accreditation) practice (33.0% ±16.2) (Table 3). Hospitals were relatively better in implementing quality systems, but poorer in the correct identification of malaria species results (Table 3; Fig. 1).

**Table 3:** Summary of laboratory performance indicators by facility type, Northeast Ethiopia, 2019 (n = 24 Health centres, eight Hospitals)

Indicator	Mean score (± SD)					
	Health centre		Hospital		Overall	
Number of tests provided	20.7	(5.6)	50.8	(15.9)	57.3	(12.9)
Percentage of standard test menu provided	53.0	(14.4)	56.4	(20.9)	54.0	(16.2)
Service functionality/uninterrupted period	99.0	(2.2)	99.5	(0.5)	99.1	(1.9)
Standard test availability period	52.5	(14.5)	56.1	(20.8)	53.6	(16.3)
Stepwise accreditation audit score	27.4	(12.9)	49.9	(13.6)	33.0	(16.2)
Concordance rate of malaria microscopy	98.6	(3.3)	97.2	(3.1)	98.2	(3.3)
Species agreement rate of malaria results	94.8	(10.1)	84.2	(25.7)	91.3	(17.1)
Concordance rate of TB microscopy	98.0	(5.2)	99.6	(0.7)	98.3	(4.7)

### Clinicians’ satisfaction level with laboratory services

Overall, the percentage of satisfied clinicians with laboratory services was 72.8% (95% CI: 69.3–76.6). In Likert scale, the overall mean (± SD) score was 3.8 (± 0.62), with mean ratings for specific aspects ranging from 3.3 to 3.9. The lowest mean ratings were obtained for the handbook’s helpfulness (3.3), provision of STAT/urgent services (3.7), and adequacy of tests provided (3.8) (Table 4).

**Table 4:** Clinicians’ satisfaction with different aspects of laboratory service, Northeast Ethiopia, 2019 (n = 224)

Satisfaction items:	Number (%)						Mean	SD	Satisfied ^*a*^, n (%)	
	Dissatisfied		Neutral		Satisfied					
Handbook’s helpfulness	59	28.7	46	22.4	100	48.8	3.3	1.1	100	48.8
Advisory service	20	9.1	32	14.5	168	76.4	3.9	0.9	168	76.4
Resolving complaint	27	12.2	22	9.9	173	78.0	3.8	0.9	173	77.9
Laboratory request form	23	10.2	19	8.5	182	81.3	3.9	0.9	182	81.3
Adequacy of test menu	22	10.0	52	23.4	148	66.6	3.8	0.9	148	66.7
Result legibility, complete	20	9.1	27	12.3	173	78.6	3.9	0.8	173	78.6
STAT/Urgent services	31	14.1	38	17.4	150	68.5	3.7	1.0	150	68.5
Clinical-lab interface	20	9.0	27	12.2	175	78.8	3.9	0.8	175	78.8
General quality of service	21	9.6	29	13.2	170	77.3	3.8	0.9	170	77.3
Overall satisfaction ^*b*^	-	-	-	-	-	-	3.8	0.6	163	72.8

^*a*^ Satisfied defined as very satisfied plus satisfied ratings (i.e., a score of ≥ 4 out of five).

^*b*^ Overall calculated based on average score from multiple (nine) satisfaction items.

**Fig. 2:**
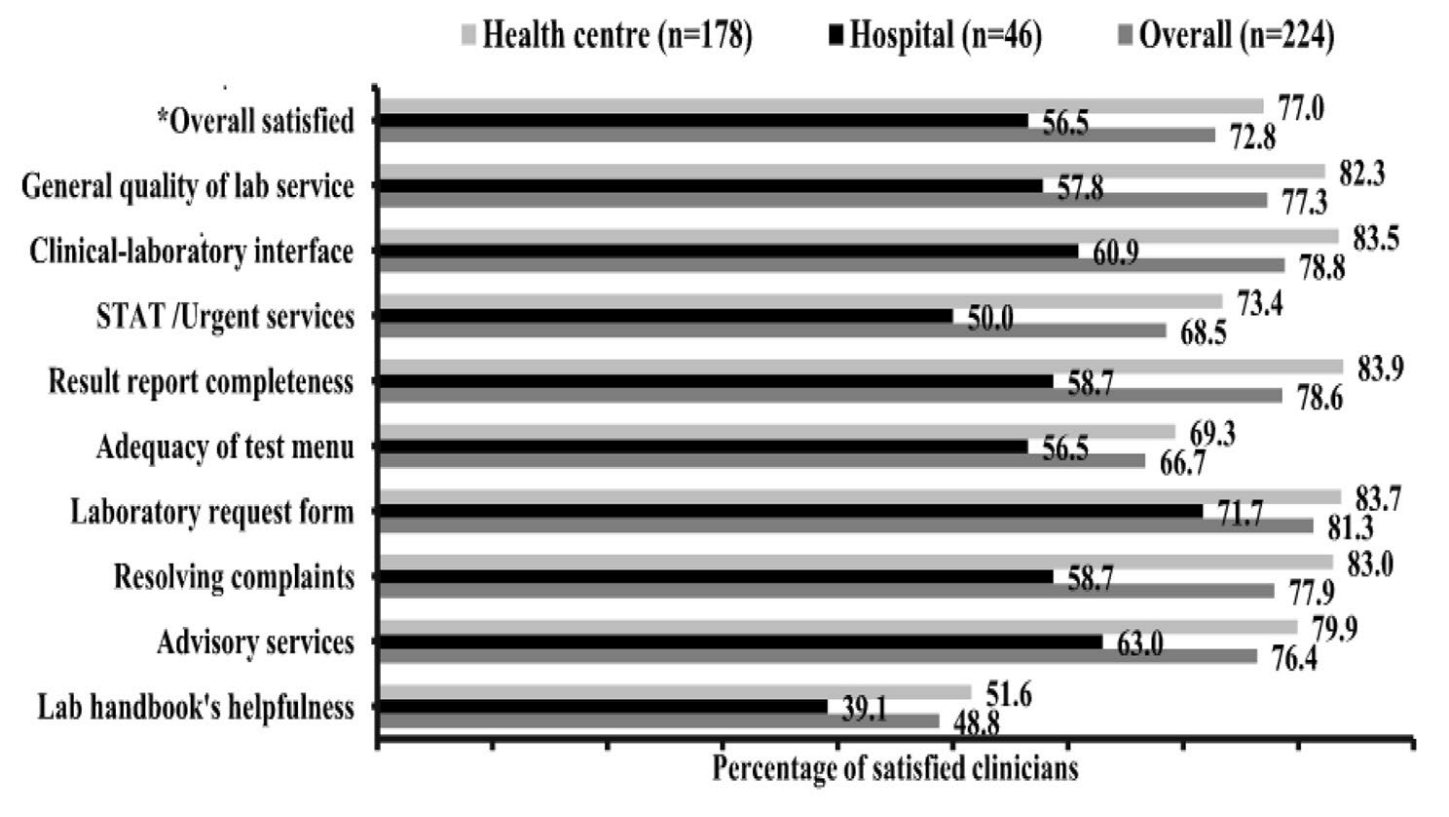
Percentages of clinicians satisfied with different laboratory service aspects by facility type, Northeast Ethiopia, 2019. *Overall satisfaction calculated at a cut-off of ≥ 4 out of five based on the average score from nine satisfaction items

The overall percentage of satisfied clinicians was relatively lower at hospitals (56.0%) compared to health centres (76.8%). Specific aspects with more dissatisfaction at the hospitals include STAT/urgent service and result completeness (Fig. 2).

### Factors associated with clinicians’ overall satisfaction

On bi-variable analysis, the clinicians’ experiences on the availability of handbook, timely communication of results, notification of panic results, quality/reliability of results and professional category, and the laboratories’ facility type, adequacy of tests, rate of concordant malaria microscopy results, and showed significant association with clinicians’ overall satisfaction (*all*, *p* ≤ 0.20). However, other variables, such as socio-demographic characteristics, and the stepwise accreditation score or star grade of the laboratories, did not show associations with clinicians’ satisfaction (*all*, *p* > 0.20) (Table 5).

**Table 5:** Association of variables with the satisfaction level of clinicians, Northeast Ethiopia, 2019 (n = 224)

Variable /Category	Satisfied ^*a*^		Not satisfied		COR	(95%CI)	*p* ^*^	AOR	(95%CI)	*p* ^*^
	n	%	n	%						
Professional category										
HO/Nurse	101	56.7	77	43.3%	1			1		
MD/Specialist	20	43.5	26	56.5%	0.6	(0.3–1.1)	0.108	0.9	(0.4–1.2)	0.939
Laboratory handbook available										
No	81	48.8	85	51.2	1			1		
Yes	37	67.3	18	32.7	2.2	(1.1–4.1)	0.019	1.3	(0.6–2.8)	0.570
Received results within claimed TAT										
No	21	29.2	51	70.8	1	(1.0–1.0)	.	1		.
Yes	91	66.4	46	33.6	4.8	(2.6–8.9)	0.000	2.3	(1.1–5.0)	0.036
Panic results communicated on-time										
No	16	27.6	42	72.4	1	(1.0–1.0)	.	1		.
Yes	102	63.4	59	36.6	4.5	(2.3–8.8)	0.000	2.5	(1.1–5.6)	0.029
Quality/reliable test results										
No	24	30.8	54	69.2	1	(1.0–1.0)	.	1		.
Yes	96	67.1	47	32.9	4.6	(2.5–8.3)	0.000	3.1	(1.5–6.3)	0.002
Backup/referral test results reliable										
No	21	29.2	51	70.8	1	(1.0–1.0)	.	1		.
Yes	88	75.9	28	24.1	7.6	(3.9–14.8)	0.000	3.9	(1.7–8.9)	0.001
Facility type/level										
Hospital	16	34.8	20	76.9	1	(1.0–1.0)		1		.
Health centre	105	59.0	83	41.9	2.7	(1.4–5.3)	0.004	1.3	(0.5–3.3)	0.588
Available test menu capacity										
Low	50	45.0	61	55.0	1			1		.
High(≥ 75%)	71	62.8	42	37.2	2.1	(1.2–3.5)	0.008	1.4	(0.7–2.9)	0.297
Concordance rate of malaria results										
Low	18	36.0	32	64.0	1		.	1		
High(≥ 95%)	103	59.2	71	40.8	2.6	(1.3–4.9)	0.004	4.1	(1.8–9.3)	0.001

^*a*^ Satisfied % calculated here as ≥ the mean score (3.8) for comparison purposes.

^*^p-value from likelihood ratio test for overall significance of variable.

COR = crude odds ratio; AOR = adjusted odds ratio; HO = health officer; MD = medical doctor.

On multivariable analysis, the clinicians’ experiences on the timely receipt of results, notification of critical results and quality/reliability of results, and the laboratories’ rate of concordant malaria diagnosis results were significantly associated with overall satisfaction (*all*, *p* ≤ 0.036). Respondents who received timely results (AOR = 2.3, 95% CI = 1.1–5.0) or were notified of panic results (AOR = 2.5, 95% CI = 1.1–5.6) were about two or three times more likely to be satisfied than their respective counterparts. Respondents who were comfortable with the quality/reliability of results (AOR = 3.1, 95% CI = 1.5–6.3) had higher odds of being satisfied compared to those who were not comfortable (Table 5).

## Discussion

In laboratory medicine, customers’ perspective has increasingly become an important tool to identify opportunities for improvement. This study assessed clinicians’ satisfaction with laboratory services delivered and associated factors at public health facilities in Northeast Ethiopia.

Overall, 72.8% of clinicians were satisfied with the laboratory services, with a mean score of 3.8 out of five. This finding is consistent with the studies conducted in eastern (3.5) [17] and southwest Ethiopia (75.0%) [18], Tanzania and Saudi Arabia (73.8-75.5%) [23–25]. The finding appears higher than studies conducted at large hospitals or among physicians in Ethiopia (51.3-62.8%) [14–16, 26, 27], and Saudi Arabia (2.7) [28]. However, the finding is lower than the findings in the USA (4.1–4.2) [6, 29], reflecting better service delivery in such developed settings.

Laboratory handbook can play an important role in communicating relevant information to users [7, 8]. In this study, the helpfulness of the handbook was the lowest-rated aspect (3.3), and most clinicians lacked a handbook (75.1%). This finding is consistent with studies where most physicians were dissatisfied with the availability or ease of understanding the handbook [14, 16, 28]. Standards require an updated guidebook that outlines the types of tests, ordering systems, types of samples and expected TATs [7, 8]. Therefore, all laboratories should provide relevant information to users through a laboratory handbook that will promote service utilization.

Timeliness of results is one of the most noticeable signs of performance that is often stressed by users [2, 6]. In this study, the second lowest-rated aspect was the timely provision of STAT/urgent tests, and clinicians who were not timely notified of panic results were more likely to be dissatisfied. These findings are consistent with many studies that have shown the dissatisfaction of most physicians with the timeliness of different test results [6, 14–16, 27, 28]. TAT targets were set for each test but not regularly monitored in most of the laboratories we assessed. Therefore, laboratories should be more sensitive to panic values and STAT requests that need urgent results for emergency or critically ill patients [2, 6]. Hospitals with high client loads may also need to consider optimizing workflows, trained couriers, or electronic clinical-laboratory interfaces to facilitate communication [6, 13].

The technical quality of laboratory results is often a point of emphasis in quality assurance frameworks. Many clinicians considered test results unreliable (35.3%), in line with findings of similar studies (39.8-87.0%) [3, 14, 16, 23, 27]. Poor perceived quality/reliability of results and a lower rate of correct malaria microscopy results were also significantly associated with dissatisfaction. Wrong diagnosis results could lead to wrong or delayed patient management and mistrust [3, 30, 31]. Thus, the technical competencies of laboratory staff or the effectiveness of existing training programs require attention [21, 30, 32, 33].

### Limitation

The first limitation is the small sample size with a 17.3% nonresponse rate, as some clinicians could not return the completed questionnaire due to workload. Second, a survey of clinician customers may not reflect the views of other laboratory customer groups. Third, there might be important variables that we did not consider, such as workload. Finally, satisfaction level can often be highly subjective and may not be fully reliable to measure actual or technical quality. However, we have tried to measure satisfaction based on multiple items and examine additional objective measures of laboratory practice.

## Conclusions

The study revealed that nearly one-third (27.2%) of the clinicians were not satisfied with the laboratory services provided. Specific areas related to both clinicians’ experiences and technical quality aspects were driving dissatisfaction. These include the absence of a helpful handbook, STAT/urgent services, communication of panic results, and reliability of test results. Therefore, laboratory managers should devise and take appropriate corrective actions to solve the root causes of the identified gaps, thereby addressing users’ needs. Particularly, emphasis should be given to improving the timely communication and quality of test results that will improve clinicians’ trust and thus utilization.

## Data Availability

All the data are available from the corresponding author up on a reasonable request.

## List of abbreviations

TAT: Turnaround time of laboratory results
TB: Tuberculosis
EQA: External quality assurance
WHO-AFRO: World Health Organization-Regional Office for Africa
ARSHB: Amhara Regional State Health Bureau
APHI-DB: Amhara Public Health Institute Dessie Branch
